# The evolution of RNAi as a defence against viruses and transposable elements

**DOI:** 10.1098/rstb.2008.0168

**Published:** 2008-10-16

**Authors:** Darren J. Obbard, Karl H.J. Gordon, Amy H. Buck, Francis M. Jiggins

**Affiliations:** 1 Institute of Evolutionary Biology, School of Biological Sciences, The University of Edinburgh, Ashworth LaboratoriesThe King's Buildings, Edinburgh EH9 3JT, UK; 2 CSIRO EntomologyGPO Box 1700, Canberra ACT 2601, Australia; 3 Division of Pathway Medicine and Centre for Infectious Diseases, University of Edinburgh Medical SchoolChancellor's Building, 49 Little France Crescent, Edinburgh EH16 4SB, UK

**Keywords:** antiviral RNAi, viral suppressors of RNAi, host–parasite coevolution, miRNA, piRNA, transposable element

## Abstract

RNA interference (RNAi) is an important defence against viruses and transposable elements (TEs). RNAi not only protects against viruses by degrading viral RNA, but hosts and viruses can also use RNAi to manipulate each other's gene expression, and hosts can encode microRNAs that target viral sequences. In response, viruses have evolved a myriad of adaptations to suppress and evade RNAi. RNAi can also protect cells against TEs, both by degrading TE transcripts and by preventing TE expression through heterochromatin formation. The aim of our review is to summarize and evaluate the current data on the evolution of these RNAi defence mechanisms. To this end, we also extend a previous analysis of the evolution of genes of the RNAi pathways. Strikingly, we find that antiviral RNAi genes, anti-TE RNAi genes and viral suppressors of RNAi all evolve rapidly, suggestive of an evolutionary arms race between hosts and parasites. Over longer time scales, key RNAi genes are repeatedly duplicated or lost across the metazoan phylogeny, with important implications for RNAi as an immune defence.

## 1. Introduction

Broadly defined, RNA interference (RNAi) constitutes a class of processes that use short RNAs (approx. 20–30 nucleotides) to recognize and manipulate complementary nucleic acids. RNAi-related pathways have roles in the control of gene expression, epigenetic modification and regulation of heterochromatin, and in the host–parasite interactions (see §2 for references). RNAi-related phenomena are so pervasive that it now seems surprising that the molecular basis of RNAi was first reported little more than 15 years ago (more that 97% of papers using the terms ‘RNAi’ and ‘RNA silencing’ have been published since 2001; for a historical perspective see Matranga & Zamore 2007). From a host–parasite and host–pathogen viewpoint, RNAi-related pathways provide a particularly interesting arena for coevolutionary interaction. For the host, RNAi is a defence against both viruses and ‘genomic parasites’ such as transposable elements (TEs), and can provide fine control over other components of innate immunity by modulating gene expression (Welker *et al*. 2007; Lodish *et al*. 2008). For the parasite, host RNAi can be exploited to subvert host cell function by manipulating host gene expression and blocking host resistance mechanisms. Strikingly, components of RNAi are widely distributed across the eukaryotic phylogeny, implying that the common ancestor of all eukaryotes had a functional RNAi pathway (Cerutti & Casas-Mollano 2006), over a billion years or more ago (Roger & Hug 2006).

Immune system genes and the parasite molecules they interact with often evolve rapidly (Schlenke & Begun 2003). It is thought that this results from a coevolutionary arms race, in which there is a reciprocal process of adaptation and counteradaptation between parasites and hosts (Dawkins & Krebs 1979). As RNAi is a primary defence of many organisms against viruses and TEs, it is likely to be a key battlefield on which these arms races are played out. This has the potential to drive the rapid evolution of the proteins involved, and ultimately shape the RNAi pathways themselves (Obbard *et al*. 2006; Aravin *et al*. 2007; Marques & Carthew 2007).

The major RNAi mechanisms have been recently reviewed (see §2) and it is not our purpose to duplicate that effort here. Instead, following a brief outline of the pathways most relevant to host–parasite biology, we review RNAi-mediated host–parasite interactions from an evolutionary perspective. We also present new analyses of the distribution of key RNAi-pathway genes across the animal phylogeny, and the rates of evolution in both RNAi genes and viral genes that suppress RNAi.

## 2. RNAi mechanisms that mediate host–parasite interactions

The RNAi-related pathways are unified by their dependence on sequence-specific binding between short (approx. 20–30 nt) RNAs and target sequences, and their common use of Argonaute family proteins. However, this belies an enormous underlying diversity between pathways and phylogenetic lineages. For example, the short RNA molecules can be derived from exogenous double-stranded RNA (dsRNA) (including viral genomes and replicative intermediates), fold-back RNAs expressed by the host or virus, or overlapping pairs of sense and antisense transcripts. Different pathways also use different complements of Dicer, Argonaute and accessory proteins, and the outcome of RNAi can be the cleavage and degradation of target RNA, the recruitment of additional factors to modify gene expression, or even long-term expression changes through epigenetic modification and heterochromatin formation. (For recent reviews see Aravin *et al*. 2007; Chapman & Carrington 2007; Ding & Voinnet 2007; Hartig *et al*. 2007; Matranga & Zamore 2007; Slotkin & Martienssen 2007; Zaratiegui *et al*. 2007; Klattenhoff & Theurkauf 2008). For a glossary of relevant terms see table 1.

### (a) The viRNA pathway

**Figure 1:**
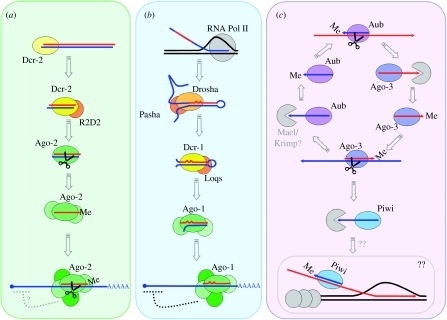
RNAi pathways in *Drosophila*. (*a*) The antiviral siRNA pathway. Dicer-2 cuts dsRNA into siRNAs, which are loaded into an Argonaute-containing RISC that targets RNA for degradation. (*b*) The miRNA pathway. Primary miRNAs are transcribed from the genome, processed by Drosha and Dicer-1 into mature miRNAs. These are loaded into the Argonaute-containing effector complex (RISC), which binds mRNAs and recruits additional factors to inhibit translation. (*c*) The ‘ping-pong’ model of TE silencing in the *Drosophila* germ line. Aubergine and Argonaute-3 (Piwi family Argonautes) alternately cleave sense (red) and antisense (blue) transcripts from TEs, guided by piRNAs generated in the other half of the cycle. Cleavage both inactivates the transcript and generates the 5′ end of a new piRNA. The new piRNA-precursor is bound by the partner Piwi family member and the 3′ end degraded and then modified by the addition of a methyl group (Me). The nuclear localization of Piwi suggests that it might mediate heterochromatin assembly (Klattenhoff & Theurkauf 2008). It is unknown how this occurs, but one possibility is that the active Piwi complex binds nascent TE transcripts to recruit heterochromatin factors (Grewal & Elgin 2007). Recently, a fourth pathway has been identified, which targets TE transcripts in both the soma and the germ line, using Dcr-2 and Ago-2 from the antiviral pathway, but Loqs from the miRNA pathway (Chung *et al*. 2008; Czech *et al*. 2008).

The best-studied role of RNAi in the immune system is as a direct defence against viruses (figure 1*a*; reviewed by Ding & Voinnet 2007). dsRNA from viruses is recognized by Dicer and cut (‘diced’) into short 21–24 nucleotide fragments called short interfering RNAs (siRNAs, also known as viRNAs when they are derived from viruses; Ding & Voinnet 2007). These are then loaded into an Argonaute-containing effector complex (RISC, the RNA-induced silencing complex; figure 1), and one strand of the viRNA is cleaved and degraded (reviewed by Tolia & Joshua-Tor 2007). The active Argonaute complex then cleaves (‘slices’) viral RNA with the complementary sequence to the viRNA (figure 1; Tolia & Joshua-Tor 2007).

In addition to this core siRNA pathway (figure 1*a*), many eukaryotes are able to amplify the viRNA pool using a host-encoded RNA-dependent RNA polymerase (RdRp), and propagate the antiviral defence signal to other cells (Chapman & Carrington 2007; Jose & Hunter 2007). In *Caenorhabditis elegans*, a host RdRp binds the siRNA–target duplex and directly synthesizes secondary siRNAs, reportedly independently of additional Argonaute or Dicer activity (Pak & Fire 2007; Sijen *et al*. 2007). In plants, a host RdRp is also recruited by the siRNA–Argonaute complex, but in this case it synthesizes long dsRNAs which are then processed into siRNAs by a Dicer family protein (Himber *et al*. 2003; Moissiard *et al*. 2007). This is thought to amplify viral-derived sequences in both plants (Schwach *et al*. 2005) and nematodes (Schott *et al*. 2005; Wilkins *et al*. 2005), and in plants RdRp knockout mutants are more susceptible to viral infection (Deleris *et al*. 2006).

In plants and nematodes, RNAi initiated in one cell can be propagated to neighbouring cells, and even to more distant tissues (‘non-cell-autonomous RNAi’; Jose & Hunter 2007). In *C. elegans*, the movement of siRNAs between cells depends on SID-1 (systemic RNAi defective 1), which is thought to be a passive channel for cell-to-cell RNA movement (Feinberg & Hunter 2003). In *Arabidopsis*, 21 nt siRNAs are transported from cell to cell for short distances (10–15 cells), and an unknown signal (probably dsRNA) is loaded into the phloem to provide long-distance transport of the silencing signal (Brosnan *et al*. 2007; Dunoyer *et al*. 2007; Dunoyer & Voinnet 2008). It has been argued that the short RNAs are likely to need to be amplified by an RdRp for the RNAi signal to be effectively propagated between cells, and in *Arabidopsis* movement beyond 15 cells is indeed dependent on RdRp function. However, many animals encode SID-1 homologues but not RdRps (see §6), and there is evidence of non-cell-autonomous RNAi in several taxa that lack their own RdRp (Jose & Hunter 2007).

### (b) The miRNA pathway

The microRNA (miRNA; figure 1*b*) pathway is a vital component of post-transcriptional control of gene expression in plants and animals (Carthew 2006; Bushati & Cohen 2007; Chapman & Carrington 2007; Pillai *et al*. 2007). MicroRNAs are encoded by the host genome and transcribed by RNA polymerase II as part of larger fold-back transcripts (primary miRNAs), which are processed in the nucleus by Drosha family members to form short stem-loops (pre-miRNAs), and then exported to the cytoplasm for processing by a Dicer family member to form the mature miRNA (figure 1*b*, reviewed by Du & Zamore 2005; Chapman & Carrington 2007; Pillai *et al*. 2007). As with viRNAs, miRNAs are loaded into an Argonaute-containing effector complex (RISC), where the complementary ‘passenger’ strand is lost. This miRNA effector complex can then interact with target messenger RNAs that contain complementary sequence to the miRNA. In plants, miRNAs are usually an exact match to their mRNA targets and cause the cleavage and degradation of the target transcript. In animals, miRNAs usually pair imperfectly with one or more targets in the 3′-UTR and do not induce mRNA endonucleolytic cleavage, but inhibit translation by a variety of mechanisms (Pillai *et al*. 2007). Although historically considered as a mechanism for the host to control its own gene expression, the miRNA pathway has more recently been shown to be important in interactions with viruses, with both hosts and viruses producing miRNAs that mediate host–virus interactions (see §4).

### (c) The piRNA and endogenous siRNA pathways

A third group of RNAi-related pathways target transposable elements (TEs: transposons, retrotransposons and endogenous retroviruses; Aravin *et al*. 2007; Hartig *et al*. 2007; Matzke *et al*. 2007; Mevel-Ninio *et al*. 2007; Slotkin & Martienssen 2007; Chung *et al*. 2008; Czech *et al*. 2008; Ghildiyal *et al*. 2008; Kawamura *et al*. 2008). It was observed in as early as 1999 that several RNAi mutants of *C. elegans* had elevated rates of transposition (Ketting *et al*. 1999; Tabara *et al*. 1999), and it is now apparent that RNAi also plays an important role in determining the activity of TEs in many other eukaryotes (Vastenhouw & Plasterk 2004; Matzke *et al*. 2007; Zaratiegui *et al*. 2007; Czech *et al*. 2008). TE transcripts are processed by Argonaute and/or Dicer family members, both reducing transcript numbers and leading to epigenetic modification of genomic copies of the transposons, heterochromatin formation and reduced expression of TEs.

In *Arabidopsis*, the pathway that targets transposons involves Dicer-like 3 and Argonaute 4, and a class of 24–26 nucleotide siRNAs derived from the TE transcripts (Matzke *et al*. 2007; Zaratiegui *et al*. 2007). In *Drosophila* and vertebrate germ lines, TE silencing relies on Argonaute proteins in the Piwi family, and a class of short RNAs known as Piwi-interacting short RNAs (piRNAs; Aravin *et al*. 2007; Hartig *et al*. 2007; Klattenhoff & Theurkauf 2008). These are longer (24–30 nt) than viRNAs and miRNAs, and originate from a single-stranded RNA (ssRNA) precursor without the involvement of a Dicer protein. In *Drosophila*, the majority of piRNAs target repeat sequences such as TEs (also known as repeat-associated siRNAs or rasiRNAs), while in vertebrates most target other repetitive sequences and only a minority are complementary to TEs. A model has been proposed in which the suppression of TEs is maintained by a cyclic feedback process that alternately cleaves sense and antisense TE transcripts (‘ping-pong’: figure 1*c*; see Klattenhoff & Theurkauf (2008) for a review).

Very recently, it has also been shown that *Drosophila* (Chung *et al*. 2008; Czech *et al*. 2008; Ghildiyal *et al*. 2008; Kawamura *et al*. 2008) and mice (Watanabe *et al*. 2008) have an additional RNAi pathway that targets endogenous dsRNA such as that produced by overlapping 3′-UTRs from genes on opposite strands, long hair-pin fold-back sequences and TEs (Chung *et al*. 2008; Czech *et al*. 2008; Ghildiyal *et al*. 2008; Kawamura *et al*. 2008; Okamura *et al*. 2008). In *Drosophila*, this pathway is similar to the antiviral pathway (figure 1*a*)—requiring both Ago-2 and Dcr-2—but it differs in that Loqs (from the miRNA pathway; figure 1*b*) takes the role of R2D2, at least for some transcripts (Chung *et al*. 2008; Czech *et al*. 2008). The resulting ‘endogenous siRNAs’ have similar size and properties to viral-derived siRNAs (viRNAs), and have a role in regulating the expression of several host genes in addition to affecting TE transcript levels.

## 3. The viRNA pathway and host–virus coevolution

Most organisms lack the adaptive immune system of vertebrates, and must rely on innate immunity to protect themselves from infection. The innate immune system often detects and destroys pathogens by recognizing families of molecules that are conserved across broad classes of pathogens (Medzhitov & Janeway 1997). In the case of bacteria and fungi, these molecules are primarily cell-surface polysaccharides, while viruses can be recognized if they produce dsRNA. dsRNA can be used to distinguish self from non-self because eukaryotes typically do not produce long stretches of dsRNA (other than that destined for processing by RNAi). However, many RNA viruses have double-stranded genomes, and even viruses with single-stranded genomes can produce dsRNA during replication, when viral RNA forms secondary structures, or when there are complementary sense and antisense replicative intermediates. DNA viruses can potentially also produce the dsRNA necessary for RNAi through secondary structure in their transcripts or sense–antisense transcript pairs (Ding & Voinnet 2007). It is now clear that the viRNA pathway (figure 1*a*) is an important component of innate antiviral immunity in plants, fungi, arthropods, nematodes and many animals.

### (a) RNAi as innate antiviral immunity

The possibility that RNAi might have an antiviral function was first raised in plants when experimentally induced ‘gene silencing’ was found to provide resistance to viruses carrying an identical sequence (Lindbo *et al*. 1993). It was then identified as a natural component of innate antiviral immunity when viruses were found to naturally induce a similar response (Covey *et al*. 1997; Ratcliff *et al*. 1997). For animals, antiviral RNAi was first identified in *Drosophila* cell culture, where the beetle Nodamura virus FHV (flock house virus) acts as both an initiator and a target of the viRNA pathway (Li *et al*. 2002). It was later confirmed as biologically relevant defence outside of cell culture using the natural host–virus combination of O'nyong-nyong virus (alphavirus; Togaviridae) and *Anopheles gambiae* mosquitoes (Keene *et al*. 2004). RNAi has subsequently been identified as a component of antiviral immunity in adult *Drosophila* (Galiana-Arnoux *et al*. 2006; van Rij *et al*. 2006; Wang *et al*. 2006; Zambon *et al*. 2006) and in nematode worms (Lu *et al*. 2005; Schott *et al*. 2005; Wilkins *et al*. 2005). Very recently, RNAi has also been identified as an important antiviral defence in fungi (Segers *et al*. 2007; Hammond *et al*. 2008). Therefore, although several isolated lineages lack components of RNAi (e.g. *Saccharomyces*), it is likely that the viRNA antiviral pathway is a shared character of most eukaryotes.

It remains an open question as to whether this pathway might be a component of antiviral immunity in vertebrates (Cullen 2006; Ding & Voinnet 2007; Haasnoot *et al*. 2007). Although vertebrates encode relevant genes (including Argonaute, Dicer and SID family members; figure 5), there is no strong evidence of a viRNA antiviral pathway, and these RNAi genes are used in other RNAi pathways (e.g. miRNA function and anti-TE function) that mediate host cellular processes.

### (b) Viral suppression and evasion of the viRNA pathway

Viral suppression of the immune system is a widespread phenomenon (e.g. Katze *et al*. 2002; Marques & Carthew 2007). It is therefore not surprising that many viruses inhibit the viRNA pathway by expressing viral suppressors of RNAi (VSRs; Li & Ding 2006; Ding & Voinnet 2007). Very soon after the natural antiviral role of RNAi was revealed, the Hc-Pro protein of plant Potyviruses (Anandalakshmi *et al*. 1998; Brigneti *et al*. 1998; Kasschau & Carrington 1998) and the 2b protein of plant cucumoviruses (Li *et al*. 1999) were identified as VSRs. The first VSR identified in an animal virus was the B2 protein of the beetle virus FHV, which was initially found to suppress RNAi in plants, before being shown to play the same role in insects (Li *et al*. 2002). As of 2006, Li and Ding were able to review the function of more than 50 VSRs from over 30 viral genera. This list includes all categories of RNA viruses (negative sense, positive sense and double stranded) and some DNA viruses, in both plants and animals. Although the VSR function of some of these genes has been questioned (especially those of negative-sense ssRNA animal viruses, e.g. Blakqori *et al*. 2007), it now appears that active suppression of the host viRNA pathways is often an important component of infection, and nearly universal in plant–RNA virus interactions (Li & Ding 2006; Ding & Voinnet 2007). VSRs appear so important for successful viral infection that several viruses encode multiple VSRs, e.g. Citrus Tristeza Virus (a Closterovirus) encodes three (Lu *et al*. 2004) and several potyviruses encode two (Valli *et al*. 2008).

Many VSRs are dsRNA-binding proteins. However, while sequestering viRNA molecules away from the RNAi pathway is apparently the primary mechanism for some—such as P19 of tombusviruses, which shows a strong specificity for 21 nt dsRNA duplexes—it is now clear that there are a diverse range of other suppression mechanisms (Li & Ding 2006; Ding & Voinnet 2007). Indeed, not all VSRs are even proteins—at least two are thought to be RNAs, which may function by binding RNAi components in place of viRNAs. Different VSRs also target different components of RNAi (Ding & Voinnet 2007). For example, both Hc-Pro and B2 appear to inhibit viRNA processing from long dsRNA precursors by Dicer. Conversely, the 2b protein of cucumoviruses and the P0 protein of poleroviruses both target Argonaute family members; 2b by binding *Arabidopsis* Ago-1 directly to prevent the RISC complex from cleaving its target RNA (Zhang *et al*. 2006*b*), and P0 by targeting Argonaute family proteins for degradation (Baumberger *et al*. 2007).

In plants, where cell-to-cell propagation of RNAi is a key feature of the viRNA pathway (and where assays for non-cell-autonomous silencing are best developed), VSRs differentially affect cell-autonomous RNAi, non-cell-autonomous RNAi and the vascular transport of RNAi (Ding & Voinnet 2007; Jose & Hunter 2007). For example, if the P38 VSR is deleted from Turnip Crinkle Virus (Tombusviridae), the virus is still able to move locally from cell to cell, but cannot spread through the entire plant (Deleris *et al*. 2006). Similarly, the Potexvirus VSR P25 does not effectively inhibit cell-autonomous RNAi, but does block long distance movement (Guo & Ding 2002). By contrast, Hc-Pro from potyviruses and AC2 from African cassava mosaic virus (a DNA geminivirus) are VSRs that suppress cell-autonomous and short-distance RNAi, respectively, but not long-distance export (Mallory *et al*. 2003; Vanitharani *et al*. 2004; Trinks *et al*. 2005). A second strategy may be for a virus to manipulate the host's own control of the pathway rather than suppressing RNAi directly. For example, the Begomovirus VSR (AC2) appears to act as a transcriptional regulator that alters the expression of approximately 50 host genes, including some that might modify RNAi activity (Trinks *et al*. 2005).

Viruses may also reduce the effects of RNAi by preventing dsRNA from coming into contact with the RNAi pathway, or avoiding degradation once viRNAs have been produced. Viruses have several adaptations to reduce the production of dsRNA and shield the viral genome from degradation (Ahlquist 2002). For example, when single-stranded viruses replicate they must produce the complementary replication intermediate, and the nucleocapsid protein of negatively stranded viruses prevents the two strands from annealing and producing dsRNA. Because viRNAs are rarely derived uniformly from across the viral genome (e.g. Molnar *et al*. 2005; Deleris *et al*. 2006), it seems likely that certain regions of the genome are more vulnerable than others. The secondary structure of RNA can determine which fragments are processed to form viRNAs, and whether the active silencing complex has access to degrade the target RNA. For example, although viroids (subviral RNA pathogens that do not encode proteins) are a source of functional viRNAs, they are not cleaved by the silencing complex owing to their secondary structure (Itaya *et al*. 2007). When HIV is cultured with an artificial RNAi construct, its secondary structure evolves rapidly to block access by RNAi components, supporting the idea that this could be a viable mechanism of viral evasion, at least in principle (Westerhout *et al*. 2005).

### (c) Viral evolution in response to host RNAi

There is clearly an evolutionary conflict between the viRNA pathway and its suppression by viruses. This has the potential to result in a never-ending arms race, where the RNAi pathway continually evolves new ways to escape suppression by VSRs, which leads to counteradaptations by the virus that restore suppression (Carthew 2006; Obbard *et al*. 2006). If this is the case, then VSRs might be expected to evolve much faster than other viral genes. This is certainly true when comparing VSRs across different viral families (Li & Ding 2006; Ding & Voinnet 2007). Li & Ding (2006) observed that VSRs from different viral families have essentially no structural similarity, even where they have similar functions. This is particularly striking when the dsRNA-binding motifs are compared across different VSRs: the dsRNA-binding domains from P19 (tombusviruses), P21 (Beet Yellows Virus) and B2 (FHV) each appear to have evolved different and entirely novel dsRNA-binding motifs (reviewed by Li & Ding 2006). Many VSRs also appear to have arisen relatively recently, and it has been argued that this is an indication of frequent viral adaptation to host RNAi (Li & Ding 2006).

If VSRs are engaged in an evolutionary arms race, then we might also expect to see the rapid sequence evolution of homologous VSRs within the same virus family. There is circumstantial evidence that this is the case (Li & Ding 2006). For example, the P1 VSR of potyviruses (Valli *et al*. 2006, 2008) is highly variable both in length and sequence, and shows evidence of recombination between potyviral lineages (Valli *et al*. 2007). Potyviruses typically have a second VSR called Hc-Pro, but in cucumber vein yellowing virus from the related genus Ipomovirus, Hc-Pro has been lost and its function appears to have been taken on by a tandemly duplicated copy of P1 (Valli *et al*. 2006). Similarly, in the Closteroviridae there is a considerable sequence diversity in the VSR P22 (Cuellar *et al*. 2008), and in one species (Sweet Potato Chlorotic Stunt Virus) some viral isolates have lost this suppressor altogether but still maintain VSR activity, presumably from a second locus (Cuellar *et al*. 2008).

**Figure 2:**
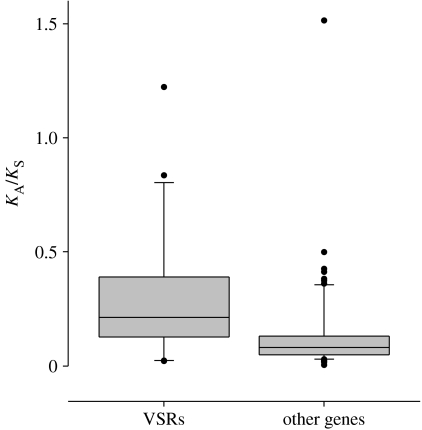
The evolutionary rate of VSRs in positive sense ssRNA plant viruses. The rate of protein evolution (*K*_A_, non-synonymous sites) relative to the rate of neutral evolution (*K*_S_, synonymous sites) for genes taken from 17 closely related pairs of ssRNA plant viruses. For viral suppressors of RNAi (VSRs) mean *K*_A_/*K*_S_=0.29 (*n*=20), and for other genes mean *K*_A_/*K*_S_=0.14 (*n*=72). In 14 of the 17 viruses, the rate of VSR evolution was higher than the average rate of the other genes (*p*<0.01, sign test). Where possible, genome pairs comprised two isolates of the same viral taxon (see the electronic supplementary material for accession details).

To test whether VSRs evolve faster than other viral genes, we analysed the rate of protein divergence for all genes in pairs of isolates from 17 ssRNA plant viruses with experimentally verified VSRs (figure 2). We found that protein divergence (relative to silent site divergence) between pairs of isolates was significantly higher for VSRs than for other genes, which is consistent with strong selection acting on VSRs. However, we would caution that the rapid evolution of VSRs could also be explained by low selective constraints (if most of the amino acid changing mutations have little effect on the function of the protein, they may be fixed at a high rate by random genetic drift), and more sophisticated analyses are needed to reject this possibility.

### (d) Subversion of the viRNA pathway

There is evidence that some viruses might have evolved to subvert the viRNA pathway for their own benefit (reviewed by Ding & Voinnet 2007). Several short RNAs derived from Cauliflower Mosaic Virus are complementary to *Arabidopsis* messenger RNAs, and downregulate those genes (Moissiard & Voinnet 2006). This may be coincidental rather than adaptive—since *Arabidopsis* is unlikely to be a natural host for cauliflower mosaic virus, this would require the short RNA targets to be very highly conserved between members of the *Brassicaceae*—but again illustrates the potential for viral evolution in response to host RNAi. It opens up interesting opportunities for the virus, as these host-manipulating viRNAs could in principle be exported from the site of infection and ‘prime’ the rest of the plant for viral invasion (Ding & Voinnet 2007).

### (e) The evolution of host viRNA-pathway genes

**Figure 3:**
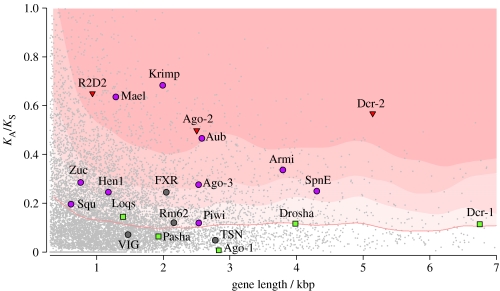
Evolutionary rate of RNAi-related genes in *Drosophila*. The rate of protein evolution (*K*_A_, non-synonymous sites) relative to the rate of neutral evolution (*K*_S_, synonymous sites) between *D. melanogaster* and *D. simulans*, plotted against aligned gene length. All 10 581 orthologous genes for which sequences are available are shown in grey, and 22 RNAi-pathway genes are shown as filled circles, squares and triangles. The smoothed median (red line) and the 75th, 85th and 95th percentiles (shaded background) are plotted for a sliding window (average 380 genes wide). RNAi-related genes evolve significantly more rapidly than the genome average (Wilcoxon signed-rank test, *p*<0.01). Six are in the fastest 5 per cent of genes (R2D2, Maelstrom, Krimper, Ago-2, Aubergine and Dcr-2), and a further 12 are in the upper 50 per cent. The piRNA pathway (purple circles) and viRNA pathway (red triangles) each evolve more rapidly than other genes (*p*<0.001 and *p*<0.01, respectively) but the miRNA pathway (green squares) does not (*p*=0.34). *K*_A_/K_S_ was calculated by the method of Li *et al*. (1985). This analysis partially duplicates that of Obbard *et al*. (2006), but uses newer genome releases and an alternative estimator of *K*_A_/*K*_S_.

Because many viruses rely on suppressing the viRNAi pathway to infect their hosts, there will be a considerable advantage to mutations in viRNAi genes that escape this suppression. Furthermore, as the VSRs themselves are evolving quickly, the viRNAi pathway faces a continually changing array of suppressors to adapt to, and this could result in an evolutionary arms race driving the rapid evolution of both host and viral proteins. As predicted by this hypothesis, three key proteins in the viRNAi pathway of *Drosophila* (Dcr-2, Ago-2 and R2D2) are among the top 3 per cent of the most rapidly evolving in the entire genome (Obbard *et al*. 2006; figure 3). Proteins involved in the immune system often have a higher rate of evolution than the genome average (Hurst & Smith 1999; Schlenke & Begun 2003), but even compared with other immunity genes the viRNAi genes evolve exceptionally fast (Obbard *et al*. 2006). Indeed, no other functional class of immunity genes shows such consistently rapid evolution. These three viRNA genes have paralogs in the miRNA pathway (Dcr-1, Ago-1 and R3D1/Loqs; figure 1), which have similar molecular functions but no direct antiviral function. The miRNA-pathway genes evolve slowly, suggesting that it is the antiviral role of viRNAi genes that is causing the high rate of evolution.

**Figure 4:**
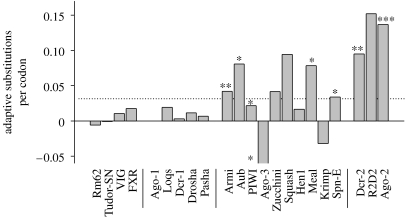
The rate of adaptive evolution in *Drosophila* RNAi genes. The estimated number of adaptive substitutions per codon (as opposed to neutral substitutions fixed by random genetic drift) for RNAi-pathway genes was estimated using the method of Smith & Eyre-Walker (2002). The analysis uses the *D. simulans* polymorphism data from Obbard *et al*. (2006; Ago-1, Ago-2, Dcr-1, Dcr-2, Loqs, R2D2) and Begun *et al*. (2007; all other genes), together with the fixed differences between these datasets and the *D. melanogaster* genome. Negative estimates (Ago-3 and Krimper) arise due to the relatively large number of amino acid polymorphisms in these genes. Genes with individually significant McDonald–Kreitman tests (McDonald & Kreitman 1991) are indicated by asterisks (^*^p<0.05, ^**^p<0.01, ^***^p<0.001, no correction for multiple tests). Note that the differences in sample size mean that the power of the test varies greatly between genes (details available in the electronic supplementary material).

However, rapid protein evolution need not be caused by positive natural selection, but can also result from low selective constraints. If a gene is evolving neutrally, the ratio of non-synonymous (amino acid changing) to synonymous differences between species will be the same as the ratio for polymorphism within species (McDonald & Kreitman 1991). In the case of the viRNAi genes, the high amino acid divergence between species is not matched by the high levels of amino acid polymorphism, providing compelling evidence that selection has driven the rapid evolution of these genes (Obbard *et al*. 2006). Using this excess of amino acid divergence relative to polymorphism, we have estimated the number of amino acid differences between *Drosophila melanogaster* and its close relative *Drosophila simulans* that were fixed because they had a selective advantage (figure 4). The rate at which natural selection has fixed selectively advantageous amino acid substitutions in the viRNAi genes is 24-fold greater than their miRNA paralogs. For R2D2 and Dicer-2 these results have also been confirmed in a second study, using a different approach that compared the genome sequences of 12 species of *Drosophila* (Heger & Ponting 2007).

## 4. The miRNA pathway and host–virus coevolution

It has recently become clear that the miRNA pathway can also play a role in host–virus interactions (Sarnow *et al*. 2006). Unlike the viRNA pathway, which primarily uses short RNAs derived from viruses as an immune defence, both host and virus sequences can be processed into miRNAs, and the miRNA pathway is not only antiviral but can also be exploited by viruses for their own benefit.

### (a) Virus-encoded miRNAs

Virus-encoded miRNAs were first identified in the Epstein–Barr virus (EBV; Pfeffer *et al*. 2004) and have now been found in at least 12 mammalian viruses, spanning the three herpesvirus subfamilies, polyomaviruses and retroviruses (for a current listing see the miRBase Sequence Database; Griffiths-Jones *et al*. 2008) (for a review see Sarnow *et al*. 2006). The miRNA pathway (figure 1*b*) provides a powerful mechanism for fine tuning gene expression in the host, and viruses make use of this pathway to modulate their own gene expression. For example, in Simian Virus 40 (SV40), the viral miRNA miR-S1 is expressed late in infection and downregulates an early viral gene that is encoded on the opposite strand of the miRNA. This autoregulation of the viral gene (the viral T-antigen) by an miRNA is proposed to be important for enabling the virus to evade the host immune response (Sullivan *et al*. 2005).

It has been estimated that more than 50 per cent of the genes in herpesviruses and poxviruses could be devoted to manipulating the host immune system (Alcami & Koszinowski 2000) and viral miRNAs are an efficient way to do this as they only require a small amount of coding sequence in the genome, yet can have a large capacity for gene regulation as they can have multiple targets. This field is in its infancy and the actual number of targets and *in vivo* functions of virus-encoded miRNAs remains to be elucidated. However, a recent report demonstrates that a miRNA encoded by Human Cytomegalovirus (HCMV) can target the MHC class I-related chain B gene (Stern-Ginossar *et al*. 2007) and thereby protect HCMV-infected cells from lysis by natural killer cells. Kaposi's sarcoma-associated herpesvirus appears to use a different strategy to manipulate host function: it encodes a miRNA (termed mir-k12-11) that mimics the host-encoded miRNA mir-155, both having the same sequence in the seed region and sharing several targets (Gottwein *et al*. 2007; Skalsky *et al*. 2007). Some of these genes regulate cell growth and B-cell transformation, so the miRNA may be partly responsible for B-cell tumours in infected patients.

Given the apparent benefits to the virus, it might be expected that many viral lineages will be found to encode miRNAs. Of the 12 viruses reported to encode miRNAs, 10 belong to the herpesvirus family, one belongs to the polyomavirus family and one to the retrovirus family. This bias is at least partly attributable to the large volume of research aimed at herpesviral miRNAs (Grey *et al*. 2008) and additional sequencing efforts will be required to understand the generality of viral-miRNA existence. However, computational analyses reveal a low probability that pre-miRNA structures occur in human and mouse ssDNA and ssRNA viruses, and cloning efforts failed to identify miRNAs in RNA viruses including Yellow Fever Virus and Hepatitis C Virus (Pfeffer *et al*. 2005). Indeed, with the exception of miRNAs recently reported from HIV, viral miRNAs have only been found in dsDNA viruses. Why might this be? First, the primary miRNA-processing machinery (e.g. Drosha) is found in the nucleus, and thus is unlikely to be available to cytoplasmic viruses. Second, host-targeted viral miRNAs must match the host sequences, and may only have time to evolve where there is a long-term host–parasite association. Third, some viruses produce VSRs that can inhibit the miRNA pathway. These factors may explain the apparent bias for miRNA representation in herpesviruses, which replicate in the nucleus, tend to remain associated with the same host lineage for millions of years (Sharp 2002), and are not known to inhibit the miRNA pathway.

### (b) Host-encoded miRNAs

Hosts can also use the miRNAs pathway as a defence against viruses by expressing miRNAs that target viral RNA (Lecellier *et al*. 2005; Pedersen *et al*. 2007). For example, Primate Foamy Virus 1 (PFV-1) mRNA is targeted by the human miRNA mir-32, which results in decreased PFV-1 replication (Lecellier *et al*. 2005). However, the physiological relevance of this might be questioned, since mir-32 may not be expressed in cell types relevant to PFV-1 replication (Cullen 2006), foamy viruses currently only occur as rare zoonoses in humans (Calattini *et al*. 2007) and it is not known whether the target sequence is conserved in related viruses that regularly infect humans. In another recent report, mice deficient in Dicer-1 were shown to be more susceptible to infection by Vesicular Stomatitis Virus (VSV) (Otsuka *et al*. 2007): two cellular miRNAs, mir-24 and mir-93, are predicted to target VSV genes encoding the viral RdRp and a polymerase cofactor, and mutation of the miRNA-binding sites in these genes rescues the growth defect in Dicer-1 deficient cells (Otsuka *et al*. 2007). It has also been suggested that miRNAs may be an effector in the classical vertebrate innate immune response (Pedersen *et al*. 2007). Activation of the interferon beta pathway in human cells infected by Hepatitis C Virus induces the expression of several host-encoded miRNAs, some of which have predicted targets in the virus (Pedersen *et al*. 2007). In support of their antiviral role, when these miRNAs are experimentally introduced they reproduce the antiviral effects of interferon beta on HCV, and the interferon defence is lost when they are experimentally removed (Pedersen *et al*. 2007). It appears that host miRNAs can also modulate cellular genes involved in the interferon response, as demonstrated for mir-146 (Taganov *et al*. 2006), and the expression of mir-146 is induced by the EBV-encoded latent membrane protein (LMP1) (Cameron *et al*. 2008), which suggests an intricate role of miRNAs in viral–host interactions.

Viruses may also exploit host-encoded miRNAs for their own advantage. For example, Hepatitis C Virus requires a host miRNA expressed in the liver, mir-122, which interacts with the 5′-non-coding region of the viral genome to increase replication (Jopling *et al*. 2005). Moreover, the host innate immune response downregulates mir-122, presumably reducing the viral replication (Pedersen *et al*. 2007).

### (c) Host–virus coevolution in the miRNA pathway

In the viRNA pathway, short RNAs are derived from the virus itself and will thus always match the target. This means that there is no possibility of the viral genome evolving to avoid sequence-based recognition. This is clearly important, as when a ‘fixed’ siRNA sequence is experimentally applied (i.e. one that is exogenous, rather than derived from the virus), the targeted viral sequence rapidly evolves to avoid being targeted (Westerhout *et al*. 2005). By contrast, in the miRNA pathway both host- and virus-encoded miRNAs may have an opportunity to coevolve with the target sequences in their opponent. As viruses generally evolve faster, thanks to their larger population sizes, shorter generation times and higher mutation rates, they may often have the upper hand.

Host-encoded miRNAs involved in the control of gene expression are often conserved over long periods of evolutionary time in both plants (Zhang *et al*. 2006*a*; Willmann & Poethig 2007) and animals (Bartel 2004; Ruby *et al*. 2007). Superficially, this might suggest that miRNA sequences are not engaged in rapid arms race evolution. However, recent sequencing of *Drosophila* miRNAs, in conjunction with analysis of 12 *Drosophila* genomes, suggests that some miRNAs are evolutionarily young and can be frequently gained and lost (Lu *et al*. 2008), opening up the possibility that there may be a class of rapidly evolving miRNAs that could target and coevolve with viruses.

The VSRs of plant viruses typically suppress the miRNA pathway as well as the viRNA pathways, and viral suppression of the host miRNA functions can be a major cost of infection (Kasschau *et al*. 2003; Chapman *et al*. 2004). Similarly, many viruses of vertebrates encode genes that suppress both RNAi pathways, possibly as a by-product of suppressing the interferon response (Cullen 2006). Given the possibility of antiviral miRNAs, it now seems plausible that this is an adaptive strategy. However, because many viruses use the miRNA pathway for their own advantage, a trade-off may occur between a virus' ability to suppress RNAi, and its ability to use miRNAs to its own advantage. Such a trade-off might not occur in *Drosophila*, where there is a separation of the core miRNA and viRNA pathways (Kavi *et al*. 2005), and VSRs do not impede the miRNA pathway *in vivo* (Chou *et al*. 2007).

## 5. Heterochromatin, the piRNA pathway, and intragenomic conflict

### (a) RNAi as a defence against TEs

TEs copy themselves throughout the genome, and can harm the host when they cause mutations or ectopic recombination (reviewed by Charlesworth *et al*. 1994). RNAi can act as a defence against TEs, both by degrading transcripts, and by preventing expression by heterochromatin formation (Grewal & Elgin 2007; Matzke *et al*. 2007; Slotkin & Martienssen 2007; Zaratiegui *et al*. 2007). Heterochromatin is usually strongly enriched for TEs (Zaratiegui *et al*. 2007), and although it is required for cellular functions such as the control of gene expression and successful chromosome segregation, it has been widely suggested that it originally evolved as an anti-TE defence (Grewal & Elgin 2007; Slotkin & Martienssen 2007; Zaratiegui *et al*. 2007).

The *de novo* initiation of heterochromatic silencing, including that targeted against TEs, seems almost universally mediated by RNAi-related pathways (Grewal & Elgin 2007; Zaratiegui *et al*. 2007), and in many organisms RNAi also plays a central role in its ongoing maintenance (Grewal & Elgin 2007). In *Arabidopsis*, more than half the endogenous siRNAs are derived from heterochromatic sequence, and a third of these target TEs and other repeats (Matzke *et al*. 2007). Here, the 24–26 nt siRNAs that target TEs are produced by a Dicer family member (DCL3), and then bound by an Ago-4-containing complex that targets the genomic copies of the TE for methylation and heterochromatin formation. A host RdRp (RDR2) is also required, suggesting that there may be an amplification step. Although it is not yet certain how this anti-TE RNAi is initiated, it has been suggested that dsRNA may be generated both by antisense TE transcripts expressed by host promoters, and by fold-back structures such as those resulting from inverted terminal repeats produced by some classes of TE (Slotkin & Martienssen 2007).

In vertebrates and *Drosophila* the piRNA pathway (figure 1*c*) appears to be a major regulator of TE activity in germ line-related cells (Aravin *et al*. 2007; Hartig *et al*. 2007). In *Drosophila* there are three Piwi-class Argonaute family proteins (Argonaute-3, Piwi and Aubergine) that are strongly expressed in the germ line. All three of these genes bind piRNAs that are primarily derived from TEs, and mutants show an elevated rate of transposition. Similarly, mutants in two of the three mouse Piwi homologues lead to increased expression of some TEs (Aravin *et al*. 2007; Carmell *et al*. 2007). Several more RNAi-related genes that are required for germ line TE silencing have been identified in the piRNA pathway of *Drosophila* (reviewed by Klattenhoff & Theurkauf 2008). The putative nucleases Zucchini and Squash are thought to be required for 3′-processing in the generation of mature piRNAs (Pane *et al*. 2007), and 3′-methylation by Hen1 stabilizes mature piRNAs (Horwich *et al*. 2007). Additionally, mutations in Spindle-E/Homeless and Armitage (which are thought to be helicases), Krimper (a tudor domain protein), and Maelstrom all disrupt piRNA formation and increase the activity of retrotransposons (Aravin *et al*. 2004; Vagin *et al*. 2006; Lim & Kai 2007).

In *C. elegans*, several RNAi-pathway genes are necessary for silencing DNA transposons in the germ line (Tabara *et al*. 1999; Sijen & Plasterk 2003), and numerous siRNAs are derived from TE sequences (Ruby *et al*. 2006). However, although Piwi family Argonautes are found in *C. elegans* and are required for germ line function, with the exception of the TE Tc3 (Das *et al*. 2008), the *C. elegans* Piwi-related genes (PRG-1 and PRG-2) do not appear to be required for the suppression of TE transposition (Batista *et al*. 2008).

Very recently, a second anti-TE RNAi pathway has been identified in *Drosophila* (Chung *et al*. 2008; Czech *et al*. 2008; Ghildiyal *et al*. 2008; Kawamura *et al*. 2008). This pathway uses Ago-2 and Dicer-2 from the viRNA pathway, and processes the TE transcripts into endogenous siRNAs in both somatic (Chung *et al*. 2008) and germ line (Czech *et al*. 2008) tissue. Although it has not yet been shown to affect rates of transposition, both Ago-2 and Dcr-2 knockouts display increased levels of TE transcript, strongly suggesting that this may be a second regulatory mechanism. Why two different RNAi pathways should be required to suppress TEs is unclear: it is possible that there is crosstalk between the pathways, and it has been hypothesized that the second pathway may be involved in the maintenance of heterochromatin in somatic tissue (Kawamura *et al*. 2008; Obbard & Finnegan 2008).

### (b) Evolution of the piRNA pathway in *Drosophila*

In the germ line, the conflict between the host genome and TEs could drive an evolutionary arms race (Charlesworth & Langley 1989), in the same way as has been hypothesized for viruses (Obbard *et al*. 2006; Marques & Carthew 2007). In support of this, a recent analysis of the genome sequences of 12 species of *Drosophila* found that natural selection has driven the rapid evolution of both Krimper and Spindle-E (Heger & Ponting 2007). To examine the other piRNA-pathway genes, we have summarized the rate of protein evolution in several components of the piRNA pathway (figure 3), and have tested these genes for effects of positive selection using the DNA sequence polymorphism data from the genome sequences of several *D. simulans* strains (Begun *et al*. 2007) (figure 4). We found that Maelstrom, Krimper and Aubergine are all among the top 5 per cent of the most rapidly evolving genes in the genome, and Armitage, Aubergine, Piwi, Maelstrom and Spindle-E show evidence of positive selection (natural selection fixing advantageous amino acid substitutions; Krimper is not significant in this test, but does appear to have an extremely high level of amino acid polymorphism within *D. simulans*). This mirrors what is seen for the viRNA-pathway genes Ago-2, Dcr-2 and R2D2, and is in contrast to the miRNA-pathways genes that evolve very slowly (see §4).

## 6. The phylogenetic distribution of RNAi components

**Figure 5:**
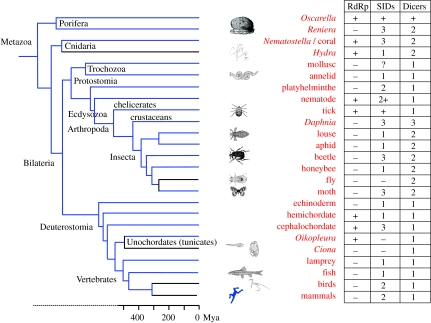
Evolutionary relationships of metazoans and their complements of selected RNAi genes. The presence of an RdRp and the number (where known) of SID and Dicer family members (Weinstock *et al*. 2006; Gordon & Waterhouse 2007) are indicated in table 1. The metazoan phylogeny is compiled from Bourlat *et al*. (2006), Delsuc *et al*. (2006) and Simionato *et al*. (2007). See figure 1 in the electronic supplementary material for sources.

While the core machinery of the RNAi pathways (figure 1) is conserved in plants and animals (Cerutti & Casas-Mollano 2006; Murphy *et al*. 2008), a fundamental difference between the kingdoms has emerged from studies of signal amplification and non-cell-autonomous RNAi. In particular, not all animal lineages carry key genes implicated in signal amplification and non-cell-autonomous RNAi (figure 5), and these differences are reflected in the diversity of RNAi phenotypes among animals (reviewed by Jose & Hunter 2007). Given the importance of signal amplification for antiviral RNAi in plants, this might be expected to have significant implications for the contribution RNAi is able to make to innate immunity in animals.

The distribution of RdRps, which are thought to be required for signal amplification, is a striking example. With extensive genome data now available from all major metazoan groups, we have been able to identify this gene in lineages on each major branch of the bilateria, consistent with its presence in the metazoan ancestor (figure 5). Although the gene has been retained in some Porifera, the Cnidaria and some of the Protostomia (e.g. nematodes and the tick) and Deuterostomia (hemichordates, cephalochordate and a tunicate), in most of these groups, it has been independently lost from at least one representative. Among the lineages known to have lost the gene are the vertebrates and insects (Wassenegger & Krczal 2006; Gordon & Waterhouse 2007; Tomoyasu *et al*. 2008), as well as the others shown in figure 5. The RdRp genes found in all animal, fungal and plant genomes are characterized by a highly conserved domain (see figure 1 in the electronic supplementary material).

SID-1, which was first identified in nematodes and forms a channel for cell-to-cell movement of dsRNA, has no orthologue in plants, but it does in many animal lineages—with the notable exception of the dipterans (figure 5). Compared to RdRp, the gene is not only more widespread, but the number of genes in each metazoan group is more variable. In the arthropods, for example, a single orthologue of SID-1 is found in each of the bee (Weinstock *et al*. 2006), louse and aphid genomes (figure 5), while multiple (three) orthologues are found in the genomes of the moth *Bombyx* and the beetle *Tribolium* (Tomoyasu *et al*. 2008). Furthermore, the relationships of the insect SIDs do not match the phylogeny of the animals they are found in. The bee SID-1 protein clusters with one of three beetle and two of three moth SID homologues (fig. 12 in Weinstock *et al*. 2006), as well as the two vertebrate SID paralogues, one of which was lost in fish, but preserved in birds and mammals. This suggests that there were ancient duplications of this gene, and some of the resulting copies have subsequently been lost in some lineages. The variation in the number of SID-1 genes may explain why the effects of RNAi seem so variable in different species. For example, if RNAi is used experimentally to knock down gene expression, it can result in systemic silencing in moths and beetles (Baum *et al*. 2007; Mao *et al*. 2007), while in flies, which have no SID-1-like genes, transgene silencing has only a localized effect. A more recent study using multiple RNAi of all SID-1 orthologues in *Tribolium* has cast a doubt on whether these genes function in systemic RNAi at all (Tomoyasu *et al*. 2008). The basis for the varying, albeit limited, extent of systemic RNAi in arthropods therefore remains unexplained. Understanding the function of the different arthropod SID genes will be of great interest, as even in *C. elegans* the function of only two of the several SID genes is known (Jose & Hunter 2007; Winston *et al*. 2007).

Dicer genes also show considerable variation among eukaryotic lineages. In plants, for example, there are larger numbers—*Arabidopsis* has four Dicer-like genes—and these show some functional redundancy (reviewed by Ding & Voinnet 2007). For example, resistance phenotypes against some viral infections are seen in *Arabidopsis* only when multiple Dicers are knocked out, suggesting that they can substitute for each other (Deleris *et al*. 2006). In *Arabidopsis*, it appears that the Dicer DCL4 is a primary effector against ssRNA viruses, but that DCL2 substitutes when DCL4 is knocked out or suppressed, and DCL3 (which normally mediates chromatin modification) plays an important role in some viral infections, and in DCL2/DCL4 double mutants (Moissiard & Voinnet 2006; Ding & Voinnet 2007). This may suggest that, in plants at least, multiple paralogous viRNA-pathway genes have specialized functions, perhaps evolving in response to viral suppression of RNAi. In animals, one or two Dicers have been found in all genomes explored to date, with the exception of *Daphnia* (figure 5; McTaggart *et al*. in press). The vertebrates and nematodes have a single Dicer responsible for both siRNA and miRNA production. Genes for two Dicers have been found in first *Drosophila*, and now in the honeybee and *Tribolium* genomes (Weinstock *et al*. 2006; Tomoyasu *et al*. 2008).

Even more striking is the variability in the components of the RISC complexes. Although it is not always clear that they function within RNAi-related pathways, Argonaute family members, in particular, seem to be very variable in number (Hock & Meister 2008; Hutvagner & Simard 2008; Murphy *et al*. 2008)—*Arabidopsis* has 10, fission yeast (*Schizosaccharomyces pombe*) 1, *Drosophila* 5, and *C. elegans* in excess of 20. Why this should be is not clear. Although there is sometimes pathway specialization (Piwi-like Argonautes function in the piRNA pathway), there is also overlap between the pathways, and genes can be functionally redundant. In the nematode, there is discrimination in loading siRNAs or miRNAs onto RISCs after processing by its single Dicer (Jannot *et al*. 2008), so that short RNAs with different functional roles are associated with different members of the large family of Argonautes. The siRNAs are found bound to RDE-1, which has RNase H activity (Yigit *et al*. 2006); by contrast, miRNAs are bound to the Argonaute proteins ALG-1 and ALG-2 (Gu *et al*. 2007). Insects and vertebrates appear to show less discrimination in this respect. In *Drosophila*, miRNAs and siRNAs are generated by distinct Dicers but the duplex intermediates undergo independent sorting processes, one involving Dcr-2/R2D2 that favours loading of siRNAs into Ago-2 (Tomari *et al*. 2007), and may be loaded into Ago-1 or into Ago-2 (Okamura *et al*. 2004; Forstemann *et al*. 2007). Ago-1 has inefficient RNase H activity (Valencia-Sanchez *et al*. 2006), so that only Ago-2 is capable of cleaving target RNAs, where there is sufficient complementarity (Zeng *et al*. 2003). In humans, which also have only one Dicer, miRNAs may be loaded onto any of the four proteins Ago-1–4; only Ago-2 has RNase H activity (Meister & Tuschl 2004).

## 7. Outlook and prospects

Despite evolution and arms races being frequently mentioned in the RNAi literature, there are still few explicitly evolutionary studies of RNAi. In this final section, we highlight three interesting topics for future work.

### (a) Specific immune responses

In this review, we have discussed how coevolution between hosts and parasites can take the form of an arms race, in which novel mutations that either increase resistance or overcome this resistance sweep through the host and parasite populations. However, coevolution can also act to maintain genetic variation in the resistance of individuals in host populations and infectivity of strains in populations (Woolhouse *et al*. 2002). This occurs because as the frequency of a resistance allele increases in the host population, the frequency of the pathogens it targets and hence its selective advantage can decrease. If the resistant allele confers susceptibility to other pathogen genotypes, or has some other fitness cost, there comes a point where it is at a selective disadvantage. An analogous process can occur within the parasite population. This results in negative frequency-dependent selection, and can maintain genetic variation in populations and promote complex dynamics, such as cycles in allele frequency.

It is possible that this form of coevolution could maintain variation in RNAi genes and viral populations. For example, because host-encoded miRNAs that target viral sequences are a form of specific immunity that is germ line encoded (as opposed to viRNAs, where immunity is acquired after infection), antiviral miRNAs will select for escape mutants in the viral population, and these escape mutants could in turn select for matching miRNAs. This could lead to specific ‘matching alleles’ in the host and viral population, in which each host allele confers resistance to some viral genotypes but susceptibility to others, resulting in the coevolutionary dynamics described above (Hamilton 1980) This could both maintain genetic variation in miRNAs and their viral targets, and lead to dynamic changes in the effectiveness of miRNA defences through time and space.

### (b) Acquired immunity

Unlike the miRNA pathway, where immunity is germ line encoded and may take many generations to evolve, highly specific viRNA-based immunity can develop rapidly after exposure to the virus. In this respect, the viRNAi pathway is reminiscent of the acquired immune response of vertebrates, which is also highly specific and develops after infection. In vertebrates, acquired immunity can maintain a high diversity of antigens in the parasite population—hosts are most likely to be immune to common antigenic types, so there is an advantage to rare antigen alleles (Hughes 1991). A similar process can occur within an infected host, where mutations in antigens that escape the immune response may be favoured, leading to a turnover of antigens through time (Allen *et al*. 2000). Could such processes occur in response to the viRNAi pathway? Plants that have been infected by a virus can develop new growth that is free from viral infection and resistant to secondary infection due to RNAi targeted against the virus (Baulcombe 2004). If these hosts are exposed to a second viral strain, then this incoming strain may have an advantage if it is not recognized by viRNAs derived from the resident strain. This could potentially maintain genetic polymorphisms in the viral population. Similarly, there may be selection for mutants that escape recognition by the viRNA pathway within an infected host, by evolving new (and thus rare) sequences (Ding & Voinnet 2007). However, the acquired immune response of vertebrates and viRNA differ in the speed of the response, whether or not it is systemic, and the number of different epitopes or viRNAs produced from a pathogen, and these factors may all affect the outcome. These ideas could be tested both theoretically and experimentally.

### (c) Why do RNAi-related genes evolve so fast?

In *Drosophila*, antiviral and anti-TE RNAi genes are among the most rapidly evolving genes in the genome (figures 4 and 5), and both viruses (Marques & Carthew 2007) and TEs (Charlesworth & Langley 1989) select for resistance in their hosts. However, while it is easy to see how the rapid evolution of antiviral genes might be driven by VSRs, it is less clear how TEs could impose such strong selection, as they are not known to suppress RNAi (although this possibility has been proposed, see Blumenstiel & Hartl 2005).

An alternative explanation for rapid evolution in the *Drosophila* piRNA pathway might be that some piRNA components also have antiviral function in addition to their anti-TE role. In support of this, some piRNA-pathway mutants appear to be more susceptible to a dsRNA virus (although these results await further experimental confirmation; Zambon *et al*. 2006), and there is an extensive overlap between the antiviral and anti-TE pathways in other organisms (e.g. Moissiard & Voinnet 2006; Brosnan *et al*. 2007). Moreover, components of the piRNA pathway might also interact with DNA viruses and retroviruses. First, DNA virus function often depends upon chromatin status (e.g. Jenkins *et al*. 2000) and in plants this can be mediated by RNAi (Bian *et al*. 2006). Second, the distinction between retroviruses and retrotransposons is a subtle one (retrotransposons are often considered ‘endogenous retroviruses’; e.g. Kim *et al*. 1994; Lecher *et al*. 1997) and in *Drosophila* retrotransposons are silenced by the piRNA pathway in the germ line (Mevel-Ninio *et al*. 2007; Pelisson *et al*. 2007), opening up the possibility that the piRNA pathway could also suppress ‘true’ retroviruses.

It is also possible that a factor other than viruses or TEs drives rapid adaptive evolution in both RNAi pathways. For example, both Ago-2 and Dcr-2 have other germ line functions in *Drosophila* (Deshpande *et al*. 2005; Jin & Xie 2007), and the Piwi family genes of both *C. elegans* (Batista *et al*. 2008) and *Drosophila* have non-TE-related germ line functions, such as mediating the silencing of other potentially ‘selfish’ sequences such as Stellate in *Drosophila* (Vagin *et al*. 2006; Aravin *et al*. 2007). Indeed, in general, the most rapidly evolving genes in *Drosophila* are those that regulate chromatin, mediate nuclear import and export, and are involved in meiosis and reproduction (Begun *et al*. 2007), and it may be that rapidly evolving RNAi genes are a part of this phenomenon. Thus, although viruses remain a prime candidate, it remains an open question as to whether viruses, TEs, other parasites, meiotic drive, sexual conflict or something else entirely has driven the adaptive evolution seen in RNAi-pathway genes.

## Figures and Tables

**Table 1:** Glossary

	term	meaning
Ago	Argonaute protein	the catalytic core of RISC that binds short RNAs and, in many cases, displays RNase H-like mRNA-cleaving activity. Key domains include the PAZ (Piwi–Argonaute–Zwille) and Piwi domains. Ago is named after an *Arabidopsis* developmental mutant that resembles the tentacles of a paper nautilus (Argonautidae)
Dcr	Dicer protein	the RNase-III family ribonuclease that cleaves dsRNA into short RNAs. Key domains include a helicase C-terminal domain, dsRNA-binding domains, a PAZ domain and two RNase-III domains. Named for its ‘dicing’ activity.
RNAi	RNA interference	the class of processes that use short single-stranded RNA molecules in complex with an Argonaute protein to bind complementary nuclei acids and modify their action and/or processing
siRNA	short interfering RNA	single-stranded RNAs of 20–30 nt involved in RNAi (especially those not classed as microRNAs)
miRNA	microRNA	single-stranded RNAs of 21–22 nt, derived from short fold-back hairpins (pre-miRNAs) and involved translational control
piRNA	Piwi-interacting RNA	single-stranded RNAs of 24–29 nt that function in complex with Piwi family Argonaute proteins in the animal germ line
viRNA	viral RNA	siRNAs derived from viral sequences
rasiRNA	repeat-associated siRNA	short RNAs derived from repeat sequences, such as TEs, sometimes considered a subclass of piRNAs in *Drosophila*
RdRp	RNA-dependent RNA polymerase	RNA polymerase directed by RNA, especially eukaryotic polymerases involved in the amplification of RNAi in nematodes and plants
RISC	RNA-induced silencing complex	the complex comprising Argonaute, a short RNA, and several other proteins, which mediates RNAi through sequence-specific complementarity
TE	transposable element	a stretch of DNA capable of moving around the genome, either by excision (cut-and-paste transposons) or through an RNA intermediate (retro-elements)
VSR	viral suppressor of RNAi	any viral gene that inhibits host RNAi function
dsRNA	double-stranded RNA	
UTR	untranslated region	non-protein-coding regions at the 5′ and 3′ ends of an mRNA
endo-siRNA	endogenous siRNA	a short RNA (other than an miRNA) that is derived from the host genome, rather than and exogenous source (e.g. a virus)

## Supplementary Material

Supplementary text 1: accession data for figure 2 Genbank accession numbers for the sequences used in figure 2

Supplementary figure 1: alignment RdRp of metazoan RdRps Alignment of sequences of RdRps from selected animal and other genomes

Supplementary table 1: numbers used for the MK tests shown in figure 4 of the main test Numbers used for the MK tests shown in figure 4 of the main test
