# Supplementary material for: The evolution of RNAi as a defence against viruses and transposable elements
Source: Philos Trans R Soc Lond B Biol Sci. 2008 Oct 16;364(1513):99–115. doi: 10.1098/rstb.2008.0168 (PMC2592633; doi:10.1098/rstb.2008.0168)
Supplement: Supplementary file 1 [file rstb20080168s02.doc]

**Supplementary material**

**Supplementary text 1: Accession data for Figure 2**

Viruses (and GenBank accession numbers) are: Turnip mosaic virus (NC_002509, AB093604), Turnip crinkle virus (NC_003821, AY312063), Tomato Bushy Stunt Virus (NC_001554, X62493) Tobacco etch virus (NC_001555, L38714), Tobacco Rattle Virus (NC_003805, AJ586803), Tobacco mosaic virus (NC_001367, X02144), Soil-borne wheat mosaic virus (NC_002041, NC_002042, AJ252152, AJ252151), Rice yellow mottle virus (NC_001575, AJ876793), Pothos latent virus (AJ243370, X87115), Potato virus Y (NC_001616, AF463399), Potato virus X, (NC_001455, EU021215), Peanut clump virus (NC_003672, NC_003668, X99149, AF239729), Grapevine virus A (NC_003604, AF007415), Cucumber mosaic virus (NC_002034, NC_001440, NC_002035, DQ399548, DQ399550, DQ399549), Beet yellows virus (X73476, AF056575), Beet mild yellowing virus (DQ132996, X83110), Barley stripe mosaic virus (NC_003469, NC_003481, NC_003478, AY789693, AY789694, AY787207
