# Supplementary material for: The evolution of RNAi as a defence against viruses and transposable elements
Source: Philos Trans R Soc Lond B Biol Sci. 2008 Oct 16;364(1513):99–115. doi: 10.1098/rstb.2008.0168 (PMC2592633; doi:10.1098/rstb.2008.0168)
Supplement: Supplementary file 2 [file rstb20080168s03.doc]

**Supplementary Figure 1: Alignment RdRp of metazoan RdRps**

Alignment of sequences of RdRps from selected animal and other genomes. The portion of the protein sequences shown corresponds to that in PF05183 at PFAM. RdRps are as follows (Genbank identifiers in brackets): *Hydra magnipapillata*: HmRdRp1, HmRdRp2 (assembled from traces - these may be polymorphisms); *C. elegans*: Ce-ego1 (AAF80367) and Ce-rrf-2 (NP_493057); Hemichordate: *Saccoglossus kowalevskii*: SkRdRp (assembled from traces); *Branchiostoma floridae*: BfRdRp (AAQ10792); anemone: *Nematostella vectensis*: NvRdRp2 (predicted from NZ_ABAV01021730 using FGENESH) and NvRdRp1 (predicted from NZ_ABAV01012549 using FGENESH) - these appear to be two distinct genes; tick: *Ixodes scapularis*: IsRdRp (predicted from ABJB010932092 using FGENESH); tunicate: *Oikopleura dioica*: OdRdRp (assembled from traces); *Arabidopsis thaliana* :At_RDR6 (NP_190519; *S. pombe*: RDR1_SCHPO (O14227). All traces were obtained from NCBI; other genomes were searched at their respective websites. The genomes marked as lacking the RdRp, apart from the well-studied insect and vertebrate genomes, were searched using BLAST and PSI-BLAST, for matches to the following RdRps: CeRdRp (NP_492132.1) and BfRdRp (AAQ10792). The RdRp of *Oscarella* is known from ESTs (EC374416.1, EC369293.1 and EC373209.1). Genomes found to lack the RdRp are as follows: Reniera: R. sp., NCBI-TraceDB; mollusc: *Aplysia californica* and *Lottia gigantea;* NCBI-TraceDB; Annelid: *Capitella capitata*; JGI; Platyhelminthe: *Schmidtea mediterranea* and *Schistosoma mansoni*, [www.genome.wustl.edu/genome_group_index.cgi](http://www.genome.wustl.edu/genome_group_index.cgi) and www.genedb.org/genedb/smansoni/; Daphnia: *D. pulex*, [www.wfleabase.org/database/](http://www.wfleabase.org/database/); echinoderm: *Strongylocentrotus purpuratus*, [www.hgsc.bcm.tmc.edu/projects/seaurchin/](http://www.hgsc.bcm.tmc.edu/projects/seaurchin/); tunicate: *Ciona intestinalis*, [www.genome.jgi-psf.org/Cioin2/Cioin2.home.html](http://www.genome.jgi-psf.org/Cioin2/Cioin2.home.html); Lamprey: *Petromyzon marinus*, NCBI-TraceDB.

```
HmRdRp1            AAKYMSRLGLYATADKETRFRISTDMISWVSDLKAKNGE-----------------LTTD
HmRdRp2            -------------ADMGKFFNISLDMCSWIDDLKAKNGE-----------------LTTD
Ce-ego-1           IPKMMARLG--QCFTQSRLSGVNLERCTYMTTYDLTGGKN----------LKGDEYTFSD
Ce-rrf-2           IPKLMARLG--QCFTQSRLTGVSLGPDNYCLTHDLSGGRS----------SNGSEYTFSD
BfRdRp             VATYVSRLG-QFFSSSRNAVSVSVED-RSVELIADVET-------------SSGTHTFSD
SkRdRp             VATYMSRMG-QCFSTSEETVNVTVED-VTVSNIEDITGGMD---------INQKPYCFSD
NvRdRp2            VAVFVSRMG-QCFSSTKKSVEVSLDNGVRIEKIPDVEKEYNNVLFPCCGNVRSGVYIFSD
NvRdRp1            VGTYVSRMG-QCFSTSLDTVGITVDG-IECLEKDDIQTE-------------EKSYTFTD
IsRdRp             VPKLMARMG-QAFSQSLGFVTVPRQYTAVEADVRCRAPPG--ASAAALAEAAKRDYIFSD
At-RDR6            VAKCAARMG-LCFSSTYATVDVMPHEVDTEVPDIERNG-----------------YVFSD
RDR1_SCHPO         VSKYAARMG-QCFSTTKEINRFCVDISLQDDIVR--NN-----------------HCFTD
OdRdRp             VSKYAARIG-LLMSPTAATIFVPDKKWAREKDIVGGPPK------------NPEKYEFTD
                                        .                                    :*

HmRdRp1            GCGKISLTMSARIANILKINQ----------PSALQIRFSGIKGMLVCT--YDDDPDLKN
HmRdRp2            GCGKVSLSMIACISKFFKIDQ----------TSALQIRFSGIKGVLVCT--NDDDPDLKN
Ce-ego-1           GVGMMSYRFAQMVSEVMDFGKG--------VPSCFQFRFRGMKGVISIEPLLDNLRQWSI
Ce-rrf-2           GVGMMSYEFAQEVSRLMSFGRS--------VPSCFQFRYRGMKGVLAIDPLLDKERNWQE
BfRdRp             GIGKISVPLAKKVAAAL---GVS------PVPSAFQIRYAGCKGVLAQDPTLGNT-----
SkRdRp             GIGKISKQLIEKICEKT---EKH------VVASAYQIRYAGSKGMVALDPSLTG------
NvRdRp2            GIGKISKDLANKVAKSLGLEAVE------MFPSAYQIRYAGCKGMLAVDPSLRG------
NvRdRp1            GIGGISSQLARKVTIKIG---KD------FIPSAFQIRYAGCKGVLAVDKSLGE------
IsRdRp             GIGRISPALMRKLEDGE-------------EPCAVQIRYGGCKGMLLVDPTMAG------
At-RDR6            GIGTITPDLADEVMEKLKLDVHy------S-PCAYQIRYAGFKGVVARWPSKSDG-----
RDR1_SCHPO         GVGMASLSVIRRLSLEVKN--HD------MFPSAFQFRMGGYKGVLSLAPPTKLEYHQG-
OdRdRp             GCGRMCTRVAEMFRDKLNLKHLYRHQGAYCVPSVIQFRMMGCKGIFVHDPTLDDADKP--
                   * *     .   .                  ..  *:*  * **:.

HmRdRp1            KP--------------LAFRPSMKKFENND------------------------------
HmRdRp2            KP--------------FAFRQSMKKFENSDKG----------------------------
Ce-ego-1           SYNISKPSDDSSWSLNCMFRPSQIKFISKRHPRDQVEIVKYSSPVPVALNKPFINILDQV
Ce-rrf-2           KHGISLSKN-----IKCVFRPSQIKFEGKQILGDQVEMVKYSSPVLVALNKPLINILDQV
BfRdRp             --------------DRIQIRGSMKKFESEHKTLEVT----SG------------------
SkRdRp             --------------EQIMIRKSMLKFPSEHDNIEIMNLTKPGE-----------------
NvRdRp2            --------------EVLQYRDSMKKFQSSHKALEIF------------------------
NvRdRp1            --------------KQLFCRPSMRKFQSSHRRLELP------------------------
IsRdRp             --------------RRIVFRTSMCKFPSDHEDLYDLKTKAR-------------------
At-RDR6            --------------IRLALRDSMKKFFSKHTILEICSWTRFQPGFLNRQIITLLSVLG--
RDR1_SCHPO         --------------NLVFPRRSQDKFKSFHSTLEVIKISRFSNAHLNMQLITLLEGLG--
OdRdRp             ---------------WVMIRDSQWKFDWNMN-----------------------------
                                      * *  **

HmRdRp1            ------------------------------------------------------------
HmRdRp2            ------------------------------------------------------------
Ce-ego-1           SEMQSLECHRRVTNRIEELLDRQMLSFAQQMVDETFCRNRLKELPRRVDIDYLRTTWGFT
Ce-rrf-2           SEMQSLECHRRITSRIEELMDLQTLSFAKQMTDEACFRNKLKEFPRRIDIDYLRTIWGFT
BfRdRp             ------------------------------------------------------------
SkRdRp             ------------------------------------------------------------
NvRdRp2            ------------------------------------------------------------
NvRdRp1            ------------------------------------------------------------
IsRdRp             ------------------------------------------------------------
At-RDR6            -----------VPDEIFWDMQESMLYKLNRILDDTDVAFEVLTASCAEQGNTAAIMLSAG
RDR1_SCHPO         ---------VEKTVFLELTRSQLSKMNESINSKQKSILMLRDNVDEYHSTLIIADFIQAG
OdRdRp             ------------------------------------------------------------


HmRdRp1            ---------------------------TTIPVENGCLVVGVADPIGILK---------EG
HmRdRp2            ---------------IYRLKVRNLLERANLSVEKGCLVVGVADPVGILK---------EG
Ce-ego-1           LSSEPFFRSLIKASIKFSITRQLRKEQIPIPCDLGRSMLGVVDETGRLQ---------YG
Ce-rrf-2           LSNEPFFRSLVKASIKFAITKQLFKEQIQIPSELGRSMLGVVDETGILQ---------YG
BfRdRp             -----------------------------------RIMMGTMDETGKLQ---------YG
SkRdRp             ---------------------LRRRARIILPPQFARNMMGVIDETGALQ---------YG
NvRdRp2            -----------------------------------------LDETGSLD---------YG
NvRdRp1            -------------------------------LESARLMMGVVDETGVLE---------YG
IsRdRp             ---------------------------ILVPPEKGRTMFGVLDETDTLE---------YG
At-RDR6            FKPKTEPHLRGMLSSVRIAQLWGLREKSRIFVTSGRWLMGCLDEAGILE---------HG
RDR1_SCHPO         FLERDDAFTENLLNLYYEWVLRLIKEKQKVSVPKGAYLLGVADETGTLKGHyDDAVLSVP
OdRdRp             ---------------------------------TSRNVYGVADISGKLR---------KG
                                                             *  . *

HmRdRp1            EIFLQIDH---------DNKKEII----------IGDALVYRNPCLHPGDQRVVCCVDKP
HmRdRp2            EIFLQIDH---------DNKKEII----------IGDALIYRNPCLHPGDQRVVCCVDKP
Ce-ego-1           QIFVQYTKNLALKLPPKNAARQVL----------TGTVLLTKNPCIVAGDVRIFEAVDIP
Ce-rrf-2           QVFVQYTKNHRNILPPRDSNRKVLG-----SEIVTGTVLLTKNPGIVPGDVRIFEAVDIP
BfRdRp             QVFIQYSREV-------DKPQADK-------VVHQGQVTVTKNPYFHPGDMRKFTAVDVP
SkRdRp             QVFIQYSKKL-------DKPQKEK-------IVHSGPVVVTKFPCYHPGDVRKFQAVDIP
NvRdRp2            EVFIRYTTEL-------GNPGQGY-------TILTGDVVVSKNPCFHPGDMRKFNAVDIP
NvRdRp1            QVFVQYSAAS-------NDPRSDKEFELSDKRVLKGPVVVTRNPCLHPGDVRRLVAIDTP
IsRdRp             QVFVQYSNDM-------FRYKATDP-----ATILKGDVIVTKNPCMHPGDIRRLTAVDVP
At-RDR6            QCFIQVSKPSIEN--CFSKHGSRFKETKTDLEVVKGYVAIAKNPCLHPGDVRILEAVDVP
RDR1_SCHPO         EIFIQITDTSTSFGSYSTGKLKTR--------VIVGLCIVARNPSLHPGDVRVCKAVRCD
OdRdRp             ECFVQITQYEEVVDKKRGTSRKERR---MLPLGNGAKVLVNKCPTYHPGDFRVLQNTHVP
                   : *::                              .   : : *   .** *

HmRdRp1            SLRYLVNVVVFPVMN-------CECSFAASCSGGDLDGDQFAIIWDTKLIPPRHLIYP-R
HmRdRp2            SLHyLVNVVVFPVIN-------CELSLAASCSGGDLDGDQFAIIWDTKLIPPRHLIYP-R
Ce-ego-1           ELHHMCDVVVFPQHG-------PRP-HPDEMAGSDLDGDEYSIIWDQQLLLDKNEDP---
Ce-rrf-2           ELHHLCDVVVFPQHG-------PRP-HPDEMAGSDLDGDEYSVIWDQKLLLERNEEA---
BfRdRp             ELRHMVDCIVFPSRG-------PRP-HPDEMSGSDLDGDMYFVTWREGLILPREN--RPA
SkRdRp             ALYHLIDCIVFPRQG-------PRP-HPNEMAGSDLDGDEYFVTWLPELLFDREN--HEA
NvRdRp2            ALYHLVDCIVFPAKG-------PRP-HTDEMSGSDLDGDKYFATWLRGIIPDRDN--EPP
NvRdRp1            KLRHLVDCVVFPKTG-------QRP-HPSEMAGGDLDGDLYFVCWYKPLIPSQEISLYDP
IsRdRp             RLHHVRDCIVFPAKG-------ERP-HPDEMSGSDLDGDEYSVLWYEDLIFRNNCS---P
At-RDR6            QLHHMYDCLIFPQKG-------DRP-HTNEASGSDLDGDLYFVAWDQKLIPPNRKS-YPA
RDR1_SCHPO         ELMHLKNVIVFPTTG-------DRS-IPAMCSGGDLDGDEYTVIWDQRLLPKIVN-YPPL
OdRdRp             ELMHLCDVIVFPVYDKVYNPDLKRP-HPHEMHESDLDGDEYFVSWDERLIPEKLEK---P
                    * :: : ::**  .        .   .     .***** :   *   ::

HmRdRp1            FDYSQLTLVDPLIKNVDVTDENVIVDFFMKFMKNDSLGRIAHKHLALCDFIEN---GARN
HmRdRp2            FDYSQLTLVDPLIKNVDVTDENVIVDFFMKFMKNDSLGRIAHKHLALCDFIEN---GARN
Ce-ego-1           YDFTSEKQKASFKE---DEIDDLMREFYVKYLKLDSVGQISNSHLHNSDQYGL---NAR-
Ce-rrf-2           FDFAVEKNLQTYEW---EDIDDLMRDVYVEYLKKDLVGLIANSHLHNSDQYGL---TSR-
BfRdRp             MDFTAQPKRVLHRPVQ----ESDMIEFFAEYIGSNQLGLIANAHLVHADKEDQ---GIFS
SkRdRp             MPFPAAVKKELTNPVE----VADMIKFVTEYIQNDCLGLMANAHLAMADQQSD---GIFS
NvRdRp2            MDFYPPKKKMLNRPIE----VSDMIQFVADYIKNDQLGVIANAHLVHADSEQD---GIFS
NvRdRp1            MDYKAPPKREDRTEIT----SADMTQYIVDYIRMDILGVIDNSHKALADKLKK---GVQS
IsRdRp             MHyYSDPPKERKAPIESVLQVQDMIDFFCQYIKGDKIGLIANAHLVWADMLDG---GINS
At-RDR6            MHyDAAEEKSLGRAVN----HQDIIDFFARNLANEQLGTICNAHVVHADRSEY---GAMD
RDR1_SCHPO         LESSPKKSIDFLEGKP---LIDSVKEFFVNYIKYDSLGLISNAWKAWAHDHDNNPEGIFG
OdRdRp             FPCPDKTIGANIKD------EITVQDLIREFTASEHLVGRINNLFIDWAKLAG----ASS
                                          : .        : :    :

HmRdRp1            PLAVELAKSQAAAVDYPKTGILPEVPKEALKLLNQH------TDINGYPDFMG---KKIS
HmRdRp2            PLAVELAKSQAAAVDYPKTGILPEVPEEACNLIKK----------NGYPDFMN---KNNS
Ce-ego-1           -VCMDLAKKNCQAVDFTKSGQPPDELERKWRKDEETGEMIPPERAERVPDYHMGNDHTPM
Ce-rrf-2           -VCMNLAKKSCQAVDFSKSGKPPDELQTTWKTDDATGEMIPPERAERVPDYHVGSDHMPK
BfRdRp             QKCIDLAQLHSDAVDAPKTGQCPELKGDLRP--------------EVYPDFMMKSD-KPQ
SkRdRp             DMCLNICKLLSEAVDFAKTGNCPTLDKNQQP--------------NTYPDFMGKQD-KTN
NvRdRp2            DKCIKLAHMHSDAVDFPKTGECPELTKELRP--------------EQYPDFMMKPD-KPR
NvRdRp1            EECLFLAEAHSYAVDAPKTGQWPDLNGLKLP--------------KSYPDFMMKTD-KPT
IsRdRp             RRCRDLARKCAVNLDFAKRGDLKGFQNSEKP--------------PMYPDFMEKLDTKNT
At-RDR6            EECLLLAELAATAVDFPKTGKIVSMPFHLKP--------------KLYPDFMGKED-YQT
RDR1_SCHPO         NVCLELAEMHSKAVDFAKSGVACKMQAKYHP--------------KRYPDFMQKTK-TRS
OdRdRp             NECRQLAKLFNQSVDTAKHGGTHNTLTPIAHN-------------------------PIF
                     .  :..     :* .* *
```
