# Supplementary material for: The evolution of RNAi as a defence against viruses and transposable elements
Source: Philos Trans R Soc Lond B Biol Sci. 2008 Oct 16;364(1513):99–115. doi: 10.1098/rstb.2008.0168 (PMC2592633; doi:10.1098/rstb.2008.0168)
Supplement: Supplementary file 3 [file rstb20080168s04.doc]

**Supplementary Table 1: Numbers used for the MK tests shown in Figure 4 of the main test.**

**polyN, fixN, polyS and fix S are the numbers of polymorphic sites in *D. simulans* and the number of fixed differences between *D. simulans* and *D. melanogaster* that are either nonsynonymous or synonymous. The number of adaptive substitutions that have occurred within each gene was estimated using the method of using the method of Smith and Eyre-Walker (2002). The Fisher’s exact test was applied to a 2 x 2 contingency table of the numbers of fixed and polymorphic nucleotide differences that were either synonymous or nonsynonymous. Also shown are the results of Heger and Ponting 2007 who tested many of these genes for positive selection (positive=significant evidence for positive selection, no=no evidence for positive selection, not in=not included in analysis)**

|  | polyN | fixN | polyS | fixS | No. of adaptive substitutions | Proportion of substitutions that are adaptive | Number of codons analyzed | Number adaptive substitutions per codon | Fishers exact test p-value | Heger & Ponting 2007 | Data Source |
| --- | --- | --- | --- | --- | --- | --- | --- | --- | --- | --- | --- |
| Ago1 | 0 | 0 | 103 | 38 | 0.0 | N/A | 851 | 0.000 | N/A | No | Obbard at al (2006) |
| R3D1/Loqs | 1 | 11 | 4 | 26 | 4.5 | 0.41 | 300 | 0.015 | 1.000 | Not in | Obbard at al (2006) |
| Dcr1 | 46 | 33 | 133 | 79 | 5.7 | 0.17 | 2036 | 0.003 | 0.500 | No | Obbard at al (2006) |
| Drosha | 6 | 25 | 44 | 72 | 15.2 | 0.61 | 1325 | 0.011 | 0.058 | No | Begun et al 2007 |
| Pasha | 3 | 7 | 38 | 34 | 4.3 | 0.62 | 642 | 0.007 | 0.312 | No | Begun et al 2007 |
| Armitage | 24 | 94 | 44 | 77 | 52.0 | 0.55 | 1265 | 0.041 | 0.007 | Not in | (derived from sequence traces) |
| Aubergine | 4 | 92 | 10 | 63 | 66.8 | 0.73 | 856 | 0.078 | 0.045 | No | Begun et al 2007 |
| PIWI | 2 | 22 | 33 | 64 | 18.1 | 0.82 | 843 | 0.021 | 0.012 | No | Begun et al 2007 |
| Ago3 | 6 | 61 | 1 | 66 | -335.0 | -5.49 | 843 | -0.397 | 0.028 | Not in | (derived from sequence traces) |
| Zucchini | 3 | 30 | 3 | 26 | 4.0 | 0.13 | 253 | 0.016 | 1.000 | No | Begun et al 2007 |
| Squash | 0 | 20 | 3 | 23 | 20.0 | 1.00 | 212 | 0.094 | 0.246 | No | (derived from sequence traces) |
| Pimet/DmHen1 | 11 | 25 | 15 | 27 | 5.2 | 0.21 | 391 | 0.013 | 0.810 | No | Begun et al 2007 |
| Maelstrom | 5 | 42 | 11 | 21 | 32.5 | 0.77 | 425 | 0.076 | 0.020 | Not in | (derived from sequence traces) |
| Krimper | 33 | 137 | 13 | 67 | -33.1 | -0.24 | 657 | -0.050 | 0.603 | Positive | (derived from sequence traces) |
| Spindle-E | 17 | 87 | 43 | 99 | 47.9 | 0.55 | 1434 | 0.033 | 0.016 | Positive | Begun et al 2007 |
| Dcr2 | 12 | 242 | 21 | 146 | 158.6 | 0.66 | 1712 | 0.093 | 0.005 | Positive | Obbard at al (2006) |
| R2D2 | 4 | 61 | 6 | 24 | 45.0 | 0.74 | 311 | 0.145 | 0.068 | Positive | Obbard at al (2006) |
| Ago2 | 4 | 115 | 5 | 5 | 111.0 | 0.97 | 817 | 0.136 | 0.000 | Not in | Obbard at al (2006) |
| Rm62 / Lip | 3 | 12 | 4 | 27 | -8.3 | -0.69 | 715 | -0.012 | 0.667 | Not in | Begun et al 2007 |
| Tudor-SN | 1 | 10 | 5 | 64 | -2.8 | -0.28 | 926 | -0.003 | 1.000 | No | Begun et al 2007 |
| VIG | 0 | 5 | 8 | 23 | 5.0 | 1.00 | 488 | 0.010 | 0.322 | Not in | Begun et al 2007 |
| FXR | 0 | 12 | 10 | 26 | 12.0 | 1.00 | 683 | 0.018 | 0.089 | No | Begun et al 2007 |
